# Evolution and bad prognostic value of advanced glycation end products after acute heart failure: relation with body composition

**DOI:** 10.1186/s12933-017-0598-3

**Published:** 2017-09-15

**Authors:** Beatriz Paradela-Dobarro, Ángel Fernández-Trasancos, Diana Bou-Teen, Sonia Eiras, Rocío González-Ferreiro, Rosa M. Agra, Alfonso Varela-Román, Ana I. Castro-Pais, Marcos C. Carreira, Felipe F. Casanueva, Ezequiel Álvarez, José R. González-Juanatey

**Affiliations:** 1 Laboratorio no. 6. Edif. Consultas externas (planta -2), Instituto de Investigación Sanitaria de Santiago de Compostela (IDIS), Complexo Hospitalario Universitario de Santiago de Compostela (CHUS), SERGAS, Travesía da Choupana s/n, Santiago de Compostela, 15706 A Coruña, Spain; 2 CIBER de Enfermedades Cardiovasculares (CIBERCV), Madrid, Spain; 3 Servicio de Cardiología y Unidad de Hemodinámica, Complejo Hospitalario Universitario de Santiago de Compostela (CHUS), SERGAS, Universidad de Santiago de Compostela, Santiago de Compostela, 15706 A Coruña, Spain; 4 División de Endocrinología, Departamento de Medicina, Complejo Hospitalario Universitario de Santiago (CHUS) and Universidad de Santiago de Compostela (USC), Santiago de Compostela, Spain; 5 CIBER Fisiopatologia de la Obesidad y Nutricion (CIBERobn), Madrid, Spain

**Keywords:** Acute heart failure, Advanced glycation end products, Soluble RAGE, Heart failure progression, Heart failure prognosis

## Abstract

**Aim:**

The role of advanced glycation end products (AGEs) and their soluble receptor (sRAGE) on the progression and prognosis of acute heart failure (HF) was analysed in relation with metabolic parameters as body composition and nutritional status.

**Methods:**

A hundred and fifty consecutive patients were included in a prospective clinical study during hospitalization by acute HF. Detailed medical history, physical examination, electrocardiogram, echocardiogram and vein peripheral blood were taken for all patients. During the follow-up period [297 days (88–422 days)] blood samples for biochemical measurements were obtained 1 and 6 months after the inclusion. Dual-energy X-ray absorptiometry analyses were performed 1 week after discharge.

**Results:**

AGEs and sRAGE levels continuously increased, up to 6 months, after acute HF, but AGEs increase was mainly observed in those patients with incident HF. Both AGEs and sRAGE levels were related with bad renal function and clinical malnutrition (CONUT score) and they were negatively related with body mass index or percentage of body fat. AGEs levels (≥40 a.u.) 1 month after discharge and basal sRAGE levels (>1000 pg/mL) were related with worse prognosis in terms of patient death and HF readmission (Log-rank <0.05 in Kaplan–Meier survival test), independently of age, gender, body mass index and other risk factors. Regression models also corroborated this finding.

**Conclusions:**

AGEs and sRAGE are bad prognostic biomarkers for HF and useful markers of HF progression. Since their levels seem to be related with clinical malnutrition and body composition these parameters could serve to modulate them.

**Electronic supplementary material:**

The online version of this article (doi:10.1186/s12933-017-0598-3) contains supplementary material, which is available to authorized users.

## Introduction

Heart failure (HF) is a leading cardiovascular problem worldwide and increasing evidence demonstrates that advanced glycation end products (AGEs) play a pivotal role in the development and progression of the disease (see for a review [1]). AGEs are generated non-enzymatically. Following their interaction with the receptors for AGEs (RAGE), a series of events leading to vascular and myocardial damage are elicited, resulting in diastolic and systolic dysfunction. Some of the molecular mechanisms involved in these events include oxidative stress, increased inflammation, enhanced extracellular matrix accumulation, and renal damage. Consequently, AGEs and soluble RAGE (sRAGE) levels are related to the severity [2, 3] and the bad prognosis [3–5] of HF, and even to the development of post-infarction HF [6].

The association between sRAGE and incident HF has been recently examined in a prospective study (The Atherosclerosis Risk in Communities Study) concluding that lower circulating levels of sRAGE are independently associated with the development of HF in a community-based population [7]. However, little is known about the evolution of sRAGE and AGEs levels during HF progression, so prospective studies are needed for this aim. Some data suggest AGEs accumulate during cardiovascular disease progression, mainly in the context of renal failure [8]. It has also been reported that the amount of AGEs in cardiomyocytes increases significantly in both diabetes and HF [9].

Evolution and progression of HF is also related with obesity and body composition. Extensive evidences demonstrate the adverse effects of obesity on central and peripheral hemodynamics, as well as on cardiac structure and function [10]. This explains the positive relationship between body mass index (BMI) and the risk of incident HF [11] or cardiovascular disease [12]. However, despite the adverse effects of obesity on cardiac structure and function, numerous studies have suggested that obese patients with HF have a better prognosis than non-obese patients, considering obesity as a category of BMI [13], a high percentage of body fat [14], or a high waist circumference [15]. This is the so-called “obesity paradox”. These data have suggested a possible protective role of body fat on HF that can be related with the fact that cardiac cachexia is related with cardiac dysfunction. All together suggests that even the weight loss recommendation usually given to obese patients with HF should be taken with care and new studies of body composition in patients with HF should be made.

AGEs could participate in the complex relationship between obesity and the outcome in patients with HF [16]. Although the underlying molecular mechanisms are far from clear some possibilities have been suggested. On one hand, the combined effects in obesity of enhanced food consumption, low-energy expenditure, hyperglycaemia, hyperlipidaemia and increased oxidative stress may boost the formation of AGEs. Moreover, AGEs could be formed during the maturation of adipocytes [17]. AGE-RAGE mediated activation may stimulate inflammatory signalling in adipose tissue, resulting in dysregulation of adipokines and contributing to the development of obesity-related complications [17]. On the other hand, AGEs are possible targets of the protective role of body fat. Gaens et al. [18] suggested that lower levels of circulating AGE (measured as carboxymethyl lysine) in obesity were associated with their trapping into the adipose tissue resulting in lower levels of circulating AGEs. This is in accordance with central obesity being characterized by chronic low grade inflammation and low levels of circulating AGEs [19]. In subjects on hemodialysis, those who naturally accumulate AGEs, the changes in skin autofluorescence (a measurement of AGEs) over 1 year period were related to BMI [20]. Accordingly, sRAGE levels, which in some conditions could act as a decoy of circulating AGEs, are inversely related with BMI in severely obese subjects [21], in young adults [22] and in subjects with metabolic syndrome, both adults [23] and adolescents [24].

Taking all these questions into account, our main objective was to analyze, for the first time, the role of AGEs and sRAGE levels in the progression of HF after an episode of acute HF decompensation in relation with other metabolic parameters like body composition, body fat distribution and nutritional status.

## Materials and methods

### Subjects

Consecutive patients (≥18 years old) admitted in our hospital by the main diagnoses of acute HF were included during hospitalization in this prospective observational study after signing informed consent. The HF diagnosis was made according to the 2016 ESC HF guidelines. If a chronic stable HF patient deteriorates it was described as “decompensated”, if it was the first admission it was considered as “de novo” [25]. Main exclusion criteria were invasive interventions within the last 6 months, acute coronary syndrome in the 3 months preceding admission, inflammatory disease, autoimmune disease and malignant disease. The whole study and protocols were approved by the Ethics Committee for Human Studies at Galicia (Spanish region) in accordance to the 1975 Declaration of Helsinki.

A complete medical history, serum biochemistry with Cobas Integra model 700 multichannel analyser (Roche Diagnostics, Indianapolis, USA) and anthropometric data were taken from all patients. Electro- and echo-cardiograms were also performed on each patient and peripheral blood samples were obtained for laboratory analysis. The diagnosis of diabetes mellitus was based on the latest criteria established by the American Diabetes Association [26]. Hypertension was defined as systolic/diastolic blood pressure >140/90 mmHg or current use of any antihypertensive medication. Dyslipidaemia was defined by total cholesterol ≥5.69 mmol/L, triglyceride ≥1.69 mmol/L, high density lipoprotein-cholesterol (HDL-C) <1.03 mmol/L, or current use of anti-hyperlipidemic drugs. Therapeutic strategy and pharmacological treatment were prescribed according to Clinical Practice Guidelines published by the European Society of Cardiology [27]. The Controlling Nutritional Status (CONUT) score was calculated for each patient at hospitalization and at discharge. The CONUT includes serum albumin, total cholesterol levels, and total lymphocyte counts, as indicators of protein reserves, caloric depletion and impaired immune defenses, respectively. Patients with CONUT scores of 0–1 have a normal nutritional status, those with CONUT scores of 2–4 are at mild risk, those with CONUT scores of 5–8 are at moderate risk, and those with CONUT scores of 9–12 are at severe risk of malnutrition [28].

### Dual energy X-ray absorptiometry (DEXA)

Body composition (fat mass percentage, fat mass and lean mass) was measured 1 week after discharge by DEXA (Prodigy, General Electric Medical Systems, Madison, WI) and the scans were analysed using enCore™ software (platform version 13.6, General Electric Medical Systems, Madison, WI). Patients were classified into low or high body fat (BF) and low or high lean mass index (LMI) attending to the criteria suggested previously by Lavie et al. [29] for both parameters. Low BF was considered ≤25% in men and ≤35% in women and high BF >25% in men and >35% in women. Regarding LMI, patients were classified into low LMI for ≤18.9 kg/m^2^ in men and ≤15.4 kg/m^2^ in women and high LMI for >18.9 kg/m^2^ in men and >15.4 kg/m^2^ in women.

### Measurements and laboratory data

Peripheral blood samples (6 mL) were taken in EDTA-anticoagulated tubes and plasma were separated by centrifugation (10 min. 1800×*g*, room temperature) and stored at −40 °C until analysis. Samples were taken at the time of discharge from the hospital and during the follow-up period at 1 and 6 months after the inclusion.

Estimated glomerular filtration rate (eGFR) was used as an indicator of renal function based on the abbreviated Modification of Diet in Renal Disease (MDRD) study formula. Cystatin C was measured by a nephelometry kit (N Latex Cystatin C; Dade Behring-Siemens Healthcare España, Madrid, Spain). AGEs were measured by quantitative fluorescence spectroscopy analysis of plasma [arbitrary units (a.u.) at 360/40:460/40 nm (excitation: emission)] following the protocol previously described [30]. By this method, we could measure some different AGE modifications at a time (crossline, fluorolink, pyrropyridine, vesperlysine, etc.), for which there are no immunological-based methods available nowadays. The intra-assay and inter-assay coefficients of variation were <4 and <10%, respectively. Plasma soluble RAGE levels were determined using a commercially available enzyme-linked immunosorbent assay kit for total soluble RAGE (DRG00, Quantikine; R&D systems, Minneapolis, MN, USA) according to the manufacturer’s protocol. Measurements were performed in duplicate and the results were averaged.

Several biochemical analytes related with diabetes and obesity were analysed in plasma at the same time by a multiplex kit (Bio-plex™, Bio-Rad, Hercules, CA, USA) using magnetic beads attached with the appropriate antibodies by a semi-automatic procedure in a Bio-Plex™ 200 System (Bio-Rad) following the instructions of the manufacturer. Adiponectin plasma levels were measured by DuoSet Enzyme-Linked ImmunoSorbent Assay (ELISA) kit with a detection limit of 0.6 ng/mL (R&D Systems, Minneapolis, MN, USA) following manufacture´s protocol.

### Follow-up and endpoints

Events were defined as either cardiac death or admission due to HF. Cardiac death was noted and confirmed by review of the death certificate, hospital chart, and physician’s records. The strong study endpoint was the combination of cardiac death and readmission by HF. The follow-up time was 297 days (inter-quartile range 188–422 days). During the observation period measurements were not determined for 26 patients at 1 month (6 of them died) and for 55 patients at 6 months (19 died) after discharge.

### Data analysis

The statistical analyses were performed with SPSS (Statistical Package for the Social Sciences), version 17.0. The categorical or dichotomous variables were expressed as absolute values and percentages, and were compared with the Pearson Ҳ^2^ test. Normality was checked with Kolmogorov–Smirnov test. The continuous variables were described as the mean ± standard deviation (SD) when normally distributed, or as the median and inter-quartile range for non-parametric data. Student *t* test was used for the comparisons of continuous variables between groups of patients (two tail distribution and equal variances between samples). Continuous data from >2 groups were compared with ANOVA followed by Tukey’s test. Non-normal distributed variables were compared with Wilcoxon text for two groups or Kruskal–Wallis test (unrelated data) or Friedman test (related data) for >2 groups comparisons. Correlations between variables were calculated by Pearson’s or Spearman’s tests according to the normality of the variable. Predictive value of individual variables for new cardiovascular events was analysed by receiver operating characteristic (ROC) curves. Different Cox proportional hazard analyses were carried out to assess the independency of AGEs or sRAGE levels to predict mortality and HF readmission and data were presented as hazard ratios (HR) with 95% confidence intervals. Kaplan–Meier curves (analyzed with log-rank test) were performed to evaluate the prognostic value of AGEs or sRAGE during follow-up. A *p* value of <0.05 was considered statistically significant.

## Results

### Baseline characteristics and laboratory data

A total of 150 consecutive patients fulfilling the inclusion/exclusion criteria were enrolled in the study from mid-2014 to mid-2015. Mean age was 69.9 ± 11.0 years and 60.7% were men. Prevalence of arterial hypertension was 77.3%, hyperlipidemia 57.3% and diabetes mellitus 46.0%. Clinical baseline parameters are presented in Table 1. Basal levels of AGEs were significantly higher in patients with New York Heart Association (NYHA) functional class IV than NYHA class II (44.1 ± 14.7 vs. 33.3 ± 6.4 a.u., *p* < 0.05). However, no differences were observed between preserved and reduced left ventricular ejection fraction (LVEF < 50%).

**Table 1:** Baseline clinical characteristics of the patients. Data presented for the total population and for the groups with or without cardiovascular events during the follow-up

	Total population (*n* = 150)	Cardiovascular event (−) (*n* = 111)	Cardiovascular event (+) (*n* = 39)	*p* value
Age (years)	69.9 ± 11.0	68.7 ± 11.4	73.4 ± 9.1	*0.024*
Male, %	60.7	57.7	69.2	0.139
HTA, %	77.3	75.7	82.1	0.280
HLP, %	57.3	51.4	74.4	*0.010*
DM, %	46.0	43.2	53.8	0.169
Smoker, %	12.7	14.4	7.7	0.323
Previous AMI, %	18.0	11.7	35.9	*0.001*
Aetiology of HF, %				
Ischemic heart disease	28.0	24.6	46.2	*0.001*
Valvular heart disease	15.3	13.5	20.5	
Hypertrophic cardiomyopathy	4.0	2.7	7.7	
Other causes	52.7	62.2	25.6	
Systolic pressure, mmHg	114.9 ± 17.2	113.8 ± 17.0	118.1 ± 17.6	0.184
Diastolic pressure, mmHg	66.4 ± 12.1	67.0 ± 11.3	64.7 ± 14.1	0.322
Laboratory data				
Glucose, mg/dL	131.5 [103.0–177.7]	126.5 [102.5–176.2]	140.0 [104.5–192.7]	0.561
NT-proBNP, pg/mL	2894 [1109–6822]	2386 [1048–4751]	6173 [1774–12723]	*<0.001*
Hb, g/dL	13.2 ± 1.9	13.3 ± 1.8	12.9 ± 2.2	0.249
HbA1c, %	6.1 [5.7–6.8]	6.1 [5.7–6.7]	6.1 [5.7–7.2]	0.430
Triglyceride, mg/dL	94 [69–121]	93 [70–124]	99 [64–120]	0.641
HDLc, mg/dL	39 [32–49]	39 [34–49]	39 [28–50]	0.432
LDLc, mg/dL	83 [63–106]	91 [68–112]	63 [50–79]	*<0.001*
eGFR MDRD, mL/min/1.73 m^2^	74.3 ± 30.0	80.1 ± 30.9	57.4 ± 19.2	*<0.001*
Echo- and electro-cardiogram data				
LVEF, %	42.9 ± 15.6	42.9 ± 14.6	42.7 ± 18.4	0.957
LV end-diastolic volume, mL	156.6 ± 71.0	150.4 ± 69.2	180.1 ± 74.5	0.096
Body composition and nutrition				
BMI, kg/m^2^	29.3 [26.1–33.3]	28.9 [25.9–34.2]	29.6 [27.1–32.5]	0.560
BF, %	37.7 ± 9.6	37.9 ± 10.0	36.9 ± 8.5	0.577
LMI, kg/m^2^	17.5 ± 3.0	17.3 ± 2.9	18.1 ± 3.1	0.204
Visceral fat, g	1878 ± 1114	1836 ± 1102	2001 ± 1158	0.459
CONUT score	2 [1–4]	2 [1–4]	3 [2–5]	0.056
Medication, %				
ACE inhibitors and/or ARBs, %	89.3	91.9	82.1	0.083
β blockers, %	78.7	79.3	76.9	0.459
Ca channel blockers, %	13.3	13.5	12.8	0.578
Statins, %	64.7	58.6	82.1	*0.029*

*ACE* angiotensin-converting enzyme, *AMI* acute myocardial infarction, *ARB* angiotensin receptor blocker, *BF* body fat, *BMI* body mass index, *CONUT* controlling nutritional status score, *DM* diabetes mellitus, *eGFR MDRD* estimated glomerular filtration rate by modification of diet in Renal Disease formula, *Hb* haemoglobin, *HbA1c* glycated haemoglobin, *HDLc* high density lipoprotein cholesterol, *HF* heart failure, *HLP* hyperlipidaemia, HTA arterial hypertension, *LDLc* low density lipoprotein cholesterol, *LMI* lean mass index, *NT-proBNP* N-terminal pro b-type natriuretic peptide

Ten biochemical parameters related to diabetes and obesity were measured by a multiplex kit: c-peptide, ghrelin, glucose-dependent insulinotropic polypeptide (GIP), glucagon-like peptide-1 (GLP-1), glucagon, insulin, leptin, plasminogen activator inhibitor-1 (PAI-1), resistin and visfatin. Adiponectin was also independently measured in plasma. Their levels were analyzed in relation with the appearance of major endpoints (Additional file 1: Table S1) and the cut-off points for AGEs and sRAGE described below (Additional file 1: Table S2). All these parameters with the exception of GIP showed a pronounced decrease from discharge to 6 months later, showing a marked relation with the acute episode of HF. These decreases were independent of the appearance of the main endpoint.

These biomarkers were also analyzed in relation with the etiology classification of HF showed in Table 1 (ischemic heart disease, valvular heart disease, hypertrophic cardiomyopathy and other causes), but no differences on these parameters were observed on this regard. Particular attention was made on PAI-1 and resistin because of their relation with inflammation, but they were no different between etiology groups at baseline, and at 1 or 6 months after discharge.

### Body composition and state of nutrition

Weight and height were measured for all patients at the moment of the inclusion and at discharge. 79.2% of the patients lost weight during hospitalization (3.55 ± 2.97 kg), whereas only 14.1% gained weight (1.41 ± 1.78 kg), the rest maintained their same weight. Body mass index was 30.3 ± 5.9 for all patients, 29.0 ± 4.8 for men and 32.4 ± 6.8 for women. Body composition of 135 patients was analysed 1 week after discharge allowing to calculate the percentage of BF and the LMI for each patient as well as the total content of visceral fat. 87.4% of the patients showed high BF. Patients with high LMI were 48.1%. Mean visceral fat was 1878 ± 1114 g, being 2077 ± 1106 g for men and 1555 ± 1060 for women.

The CONUT score showed that at the time of hospital admission 20.6% of patients were at normal, 54.0% at mild, 21.3% at moderate and 1 patient at severe risk of malnutrition. At the time of discharge, 32.0% were at normal, 50.0% at mild, 14.7% were at moderate and nobody at severe risk of malnutrition. In summary, 23.3% of patients reduced their malnutrition risk and only 4.6% increased it during hospitalization.

### AGEs and sRAGE

**Fig. 1:**
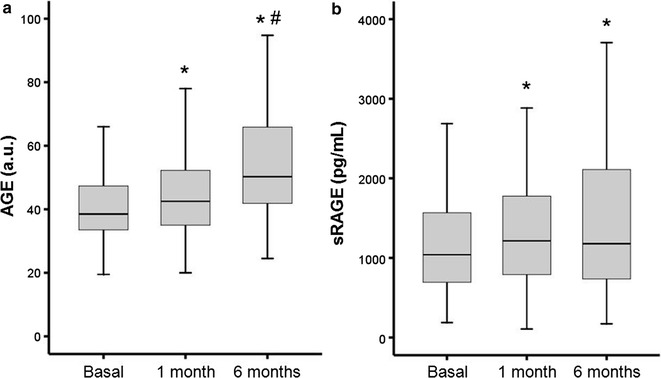
AGEs (**a**) and sRAGE (**b**) plasma levels at time of discharge and after 1 or 6 months. Boxes represent interquartile ranges with median as horizontal line. Vertical bars demarcate the maximum to minimum range. *p <0.05 vs. basal values, # <0.05 vs. 1 month levels, by Wilcoxon test

AGEs and sRAGE levels were measured at discharge (AGE_0_ and sRAGE_0_) and 1 (AGE_1m_ and sRAGE_1m_) and 6 months (AGE_6m_ and sRAGE_6m_) after discharge. The results of these measurements are shown in Fig. 1 and a progressive and significant increase in AGEs and sRAGE levels is observed up to 6 months after the acute HF. However, this progressive increase depends on the previous history of HF, such that, patients included in the study with incident HF (64% of total) showed significant increase in levels at AGE_1m_ and AGE_6m_, but not in sRAGE levels, although some trend was observed (*p* = 0.074 and *p* = 0.064 for sRAGE_1m_ and sRAGE_6m_ with respect to sRAGE_0_, respectively; Additional file 1: Figures S1a, S1b). On the contrary, in patients with previous HF sRAGE levels progressively increased up to 6 months, whereas AGEs levels only increased after 6 months of discharge. In a similar way, previous acute myocardial infarction (AMI) induced higher increase in sRAGE_1m_ than no previous AMI, but it seems not to influence the evolution of AGEs levels (Additional file 1: Figures S1c, S1d). Analyzing by the appearance of the main endpoint (death + HF readmission) there were no statistical differences in the evolution of AGEs or sRAGE levels, although both parameters were slightly higher in the group with events (Additional file 1: Figures S1e, S1f).

Direct association between the CONUT score and the levels of sRAGE_0_ and AGE_1m_ were observed. The levels of these variables positively increased when risk of malnutrition increased (Additional file 1: Figure S2).

AGEs and sRAGE levels showed an inverse relationship with several parameters of obesity. Basal AGEs negatively related with BMI (*r*_*s*_ = −0.175; *p* = 0.037) and sRAGE_1m_ levels were negatively related with BF percentage (*r*_*s*_ = −0.259; *p* = 0.014). AGEs_1m_ and sRAGE_1m_ were lower in the highest quartile of BF percentage than in the lowest (38.2 [33.2–50.0] vs. 43.4 [39.2–53.8] a.u., *p* = 0.038 for AGEs_1m_; 1017.0 [780.5–1577.0] vs. 1661.0 [898.0–2843.0] pg/mL, *p* = 0.041 for sRAGE_1m_). Soluble RAGE_1m_ were also lower in obese (BMI > 30 kg/m^2^) than “normal” BMI patients (BMI < 25 kg/m^2^; 1043.0 [702.7–1588.5] vs. 1603.5 [1183.7–2613.2] pg/mL, *p* = 0.022). Bad renal function measured by creatinine levels was related with both AGE_1m_ (*r*_*s*_ = 0.315; *p* = 0.001) and sRAGE_1m_ levels (*r*_*s*_ = 0.267; *p* = 0.008). Even more, AGE levels (at all times) and sRAGE_1m_ levels were significantly higher in the group of patients with eGFR <60 mL/min/1.73 m^2^ (39.7 [34.5–47.9] vs. 51.0 [40.0–63.7] a.u., *p* = 0.001 for AGE_1m_ and 982.0 [673.0–1444.0] vs. 1081.0 [697.0–1737.0] pg/mL, *p* = 0.004 for sRAGE_1m_). This relationship with renal function is well known and established.

### Follow-up data

**Fig. 2:**
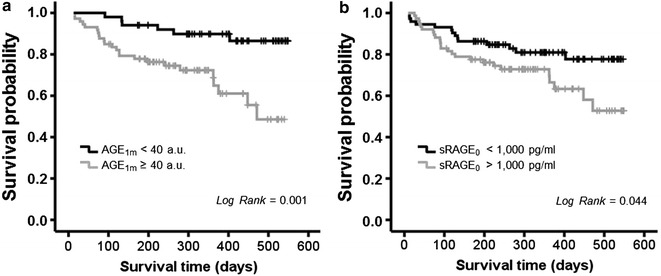
Cumulative survival curves during the follow-up period for patients grouped accordingly to the AGE levels at 1 month of discharge (AGE_1m_) by the cut-off point of 40 a.u. (**a**) or by the cut-off point of 1000 pg/mL of basal sRAGE (sRAGE_0_) concentration (**b**). Survival was considered free of death or HF readmission. Kaplan–Meier curves were analysed with log-rank test and their *p* value is showed

The value of AGE and sRAGE levels to predict new events (defined by the combination of death and HF readmission) after acute HF were analysed by receiver operating characteristic (ROC) curves. A total of 39 patients suffered an event during the follow-up period and the clinical parameters for this group in comparison with event-free patients are presented in Table 1. AGE_1m_ was the one that presented a better area under the curve of the three values of AGE levels [0.613 95% CI (0.499, 0.727)]. From that curve we obtained a cut-off value of 40 a.u. AGE_1m_ (0.8 sensitivity, 0.5 specificity) for predicting death and HF readmission. Interestingly, AGE_1m_ levels were positively related with NT-proBNP levels (a known biomarker of HF). sRAGE_0_ showed an area under the ROC curve of 0.621 [95% CI (0.516, 0.725)] and from that curve we selected a cut-off value of 1000 pg/mL (0.6 sensitivity, 0.5 specificity). These cut-off values for AGE_1m_ and sRAGE_0_ served to predict worse prognosis of patients in terms of death and HF readmission (Long-rank < 0.05 in Kaplan–Meier survival tests; Fig. 2). Multivariate analyses by Cox regression models also corroborated this finding. The best regression model was for the variables presented in Table 2. Presence of hyperlipidaemia (HLP), NT-proBNP levels (measured in 100 pg/mL steps) and more than 40 a.u. of AGE_1m_ were predictors of cardiovascular events (considering death or HF readmission) after acute HF, adjusted by age, gender, diabetes mellitus, eGFR <60 mL/min/1.73 m^2^, sRAGE levels and LMI.

**Table 2:** Multivariate analysis for AGE to predict follow-up mortality and HF readmission after discharge for acute HF

Multivariate model (adjusted by age, gender, eGFR_60_, diabetes mellitus and LMI)			
Variable	HR	CI 95%	*p*
HLP	2.870	1.145–7.195	*0.025*
NT-proBNP_100_	1.006	1.002–1.010	*0.007*
AGE_1m_ > 40 a.u.	2.775	1.080–7.130	*0.034*
sRAGE_0_ > 1000 pg/mL	2.064	0.901–4.728	0.086

*eGFR*_*60*_ estimated glomerular filtration rate <60 mL/min/1.73 m^2^, *HLP* hyperlipidaemia, *LMI* Lean mass index, *NT-proBNP*_*100*_ N-terminal pro b-type natriuretic peptide in 100 pg/mL steps

Values in italics denote statistically significant differences

Basal biochemical parameters showed no differences between event- and event-free groups, unless for GLP-1 and glucagon, both being significantly higher in the event-group (Additional file 1: Table S1). Regarding the cut-off predicting values of AGE_1m_ and sRAGE_0_ only basal levels of c-peptide and resistin were higher when AGE_1m_ > 40 a.u. whereas PAI-1 was lower. In the case of sRAGE_0_ >1000 pg/mL only basal insulin was significantly lower (Additional file 1: Table S2).

## Discussion

In this work, the evolution of AGEs and sRAGE levels during the 6 months following an acute HF episode of decompensation is studied for the first time. AGEs and sRAGE levels continuously increased in this period, but the increase of AGEs was mainly observed in those patients with incident HF. This relation with the evolution of HF is a novel finding of this work. It was also confirmed that sRAGE levels are predictive biomarkers of bad HF prognosis independent of age, gender, body mass index and other risk factors, in a 1-year follow-up period. Interestingly, high AGE_1m_ levels after the acute decompensation are also indicative of a worse clinical outcome in terms of cardiovascular death and HF readmission. Finally, AGEs and sRAGE levels showed a direct association with clinical malnutrition (CONUT score), but an inverse relation with BMI or the percentage of body fat.

Basal levels of AGEs were related to the severity of the HF in terms of NYHA functional classes at the time of hospital admission, but not in relation with preserved or reduced ejection fraction. On the contrary, sRAGE did not show this relationship with HF severity. These results are different than those previously observed in patients with chronic HF, where sRAGE levels correlated with NYHA class [2, 5]. However, it should be taken into account that in the present study we are analyzing patients in the moment of the acute decompensation by HF, most of them in class III and IV, and that 64% of these patients are cases of incident HF. In the previously mentioned studies the patients presented chronic and stable HF with a more homogeneous distribution over the NYHA classes. In this sense, the influence of the inflammatory status of each patient in the moment of sampling could affect AGEs or sRAGE levels. However, the levels measured in this work for PAI-1 and resistin, two cytokines that have been related with the sub-clinical inflammatory status [31, 32], were no different regarding the cutoff point of sRAGE_0_, and they were contradictory regarding the cutoff point of AGE_1m_: whereas resistin was higher in the group of AGE_1m_ > 40 a.u., PAI-1 was lower.

Moreover, we did not observe differences in sRAGE levels between patients with preserved or reduced LVEF as Willensen et al. [33] did, but we considered reduced LVEF as <50% whereas they used <40%. A possible explanation for these differences could arise from one of the novel findings of this work: AGEs and sRAGE levels increase during the development of HF. At least during 6 months following an acute decompensation by HF, both biomarkers continuously increased their values, being this event more evident for AGEs in the case of incident HF. Therefore, the levels of these biomarkers seem to be influenced by the stage of the HF. Our data show that AGEs levels increase noticeably after an acute episode of incident HF, whereas sRAGE seems to react in the same way, but with a slower rate, which is why the increase is better observed in patients with previous HF. This means that AGEs and sRAGE levels are not only biomarkers of the severity of HF, but also of its progression. This is an important point since the rest of the biomarkers measured in the study, all related with diabetes and obesity, showed the opposite relationship with acute HF. They all presented a marked reduction from their elevated levels during hospitalization, to lower levels during the months following the discharge.

AGE-RAGE axis seems to be related to the nutritional status of patients since CONUT scores indicating risk of malnutrition were directly associated with AGE and sRAGE levels. This is an interesting and new finding, which is in accordance with the inverse relationship observed between AGE and sRAGE levels with respect to parameters of obesity such as BMI or BF percentage. We previously commented that sRAGE levels were inversely related to BMI in severely obese subjects [21], in young adults [22] and in subjects with metabolic syndrome either adults [23] or adolescents [24]. More recently, the inverse relationship between sRAGE and BMI was observed in healthy women [34]. Soluble RAGE levels were also inversely associated with fat mass, and in patients with prediabetes [35], where it also was negatively correlated with body weight and waist and hip circumferences. As we will discuss below, high sRAGE levels are indicators of bad prognosis in the population under study. Therefore, these findings support the hypothesis of the so-called “obese paradox”, by which obesity and particularly body fat, have shown to be protective in the context of HF [14]. In this case, high BMI and BF correlate with lower sRAGE levels, predicting a better outcome than in patients with higher sRAGE levels and lower BMI and BF.

The relationship between AGEs and obesity seems to be more complex. According to previous data [17], AGEs accumulation in adipose tissue could explain our findings about the inverse relationship between AGEs levels and BMI, which agrees with previous results [18, 19], but disagrees with Amin et al. [36], that found a positive relation between AGEs, measured as carboxymethyl lysine, and BMI. Although the mechanisms are controversial, AGEs can be absorbed from diet. In this sense, whereas low-AGE diets can increase insulin sensitivity [37], high-AGE diets are related with increased fat intake and higher risk of abdominal obesity [38]. However, metabolic syndrome can make the difference: serum AGEs levels and AGEs consumption from diet are higher in obese people with metabolic syndrome than in obese without metabolic syndrome [39]. Once in the body, RAGE mediates AGE accumulation in adipose tissue [18], and RAGE activation can induce the production of inflammatory mediators in adipocytes that could ultimately lead to dysregulation of adipokines in obesity [17].

Present data confirm previous findings about the predictive value of AGE-RAGE axis for worse prognosis in HF for AGEs [3, 6] and sRAGE [4, 5] levels. Calculated cut-off values for AGE_1m_ and sRAGE_0_ served to predict death and HF readmission in the univariate analyses. The best regression model included hyperlipidemia, NT-proBNP levels and more than 40 a.u. of AGE_1m_ as predictors of death or HF readmission after acute HF, adjusted by age, gender, renal function, diabetes mellitus and LMI. These results partially agree with those recently reported by Willensen et al. [33] where AGE predicted hospitalization for HF and the combination of mortality and HF hospitalization whereas sRAGE did not predict events with statistical signification. Apart from the direct molecular damage of the activity of AGE-RAGE axis in cells, tissues and organs of the cardiovascular system [1], high AGE levels in plasma severely affect kidney function. This damage is always reflected by the positive correlation between AGE and sRAGE levels and parameters indicating bad renal function. Renal impairment is common in HF, and strongly associated with poor outcome [40], so this could be one reason for the bad prognostic value of AGE and sRAGE in this study.

Another possible reason is the relation with energetic metabolism and obesity in our population. We have previously commented the relationship between AGE and sRAGE levels with malnutrition. Moreover, high AGEs levels were related to higher concentrations of c-peptide and resistin in plasma and to lower concentration of PAI-1, suggesting some relation with the modulation of insulin secretion and insulin resistance for AGEs. PAI-1 can respond to increased inflammation and fibrosis, but also to obesity or metabolic syndrome [41]. Intriguingly, low levels of insulin were observed in patients with higher levels of sRAGE. The group of patients with events showed higher levels of glucagon and GLP-1, suggesting an enhanced gluconeogenesis and aside from this, a possible implication of increased energy expenditure and decrease of adipose tissue expansion in this group of patients [42].

Finally, although do not directly supported by our data, we cannot forgot that AGEs have been demonstrated to participate in the structural modification and functional alteration of the extracellular matrix proteins in vessels and myocardium, as well as in the intracellular signals mediated by calcium in cardiovascular cells [1, 43]. All these mechanisms would also contribute to explain the pathophysiological role of AGE/RAGE axis on HF.

### Conclusions

As the conclusion of the work, both AGEs and sRAGE seem to be fine biomarkers of bad prognosis for HF. However, the main novelty is related with the evolution of AGE/RAGE axis in relation with HF. AGEs and sRAGE levels increase at the beginning of the pathology, being useful for following HF progression. Since this increase seems to be related with clinical malnutrition and negatively correlated with body fat quantity, these parameters could serve to modulate their levels in a trend to improve HF clinical outcome.

### Limitations

The main limitation of our study is the number of patients included. This reduces the potency of the statistical analysis. Further research in large populations is needed to confirm our findings. Secondly, our study was made only in patients after an acute episode of HF, so, the results can be different in patients with chronic and established HF. Thirdly, the culprit mechanism of the acute episode of HF is unknown in most of the patients, so differences between patients with regard to this could not be explored. Fourthly, sampling during the follow-up occurred at only three time points, hence it is not possible to know what happens in the periods in between. Fifthly, AGE measurement was done for a group of modifications, as explained, not for all the types of AGEs or for only a specific AGE, so, 1) the results cannot be extrapolated to all the AGEs and 2) the specific effect of one single AGE of the type of AGEs measured cannot be extracted from the whole.

## Additional file

**Additional file 1: Table S1.** Basal levels of biochemical parameters analysed. Data are presented as median [interquartile range] for the total population and grouped by the presence of events (death or HF hospitalization). **Table S2.** Basal levels of biochemical parameters analysed. Data are presented as median [interquartile range] for the total population and grouped by the levels of AGE_1m_ and by sRAGE_0_. **Figure S1.** Influence of previous heart failure (HF, **a**, **b**), previous acute myocardial infarct (AMI, **c**, **d**) or incidence of cardiovascular events (death or HF hospitalization, **e**, **f**) in the plasma levels of AGEs (**a**, **c** and **e**) and sRAGE (**b**, **d** and **f**) at time of discharge and after 1 or 6 months. Boxes represent interquartile ranges with median as horizontal line. Vertical bars demarcate the maximum to minimum range. **p* < 0.05 vs. basal values, #< 0.05 vs. 1 month levels, by Wilcoxon test. **Figure S2.** Levels of AGE at 1 month after discharge (**a**) and of sRAGE at discharge (**b**) in relation with the CONUT score at discharge. Boxes represent interquartile ranges with median as horizontal line. Vertical bars demarcate the maximum to minimum range. **p* < 0.05 by Kruskal–Wallis test.
