# Supplementary material for: Evolution and bad prognostic value of advanced glycation end products after acute heart failure: relation with body composition
Source: Cardiovasc Diabetol. 2017 Sep 15;16:115. doi: 10.1186/s12933-017-0598-3 (PMC5602867; doi:10.1186/s12933-017-0598-3)
Supplement: Supplementary file 1 — Additional file 1: Table S1. Basal levels of biochemical parameters analysed. Data are presented as median [interquartile range] for the total population and grouped by the presence of events (death or HF hospitalization). Table S2. Basal levels of biochemical parameters analysed. Data are presented as median [interquartile range] for the total population and grouped by the levels of AGE1m and by sRAGE0. Figure S1. Influence of previous heart failure (HF, a, b), previous acute myocardial infarct (AMI, c, d) or incidence of cardiovascular events (death or HF hospitalization, e, f) in the plasma levels of AGEs (a, c and e) and sRAGE (b, d and f) at time of discharge and after 1 or 6 months. Boxes represent interquartile ranges with median as horizontal line. Vertical bars demarcate the maximum to minimum range. *p < 0.05 vs. basal values, #< 0.05 vs. 1 month levels, by Wilcoxon test. Figure S2. Levels of AGE at 1 month after discharge (a) and of sRAGE at discharge (b) in relation with the CONUT score at discharge. Boxes represent interquartile ranges with median as horizontal line. Vertical bars demarcate the maximum to minimum range. *p < 0.05 by Kruskal–Wallis test. [file 12933_2017_598_MOESM1_ESM.doc]

**Evolution and bad prognostic value of advanced glycation end products after acute heart failure: relation with body composition**

Beatriz Paradela-Dobarro^1,2^, Ángel Fernández-Trasancos^1^, Diana Bou-Teen^1^, Sonia Eiras^1,2^, Rocío González-Ferreiro^3^, Rosa M. Agra^1,2,3^, Alfonso Varela-Román^1,2,3^, Ana I. Castro-Pais^4,5^, Marcos C. Carreira^5^, Felipe F Casanueva^4,5^, Ezequiel Álvarez^1,2,*,#^, José R. González-Juanatey^1,2,3,#^.

^#^ These authors equally contributed to the work.

1. Instituto de Investigación Sanitaria de Santiago de Compostela (IDIS). Complexo Hospitalario Universitario de Santiago de Compostela (CHUS). SERGAS. Travesía da Choupana s/n, Santiago de Compostela, 15706 A Coruña, Spain.

2. CIBER de Enfermedades Cardiovasculares (CIBERCV), Madrid, Spain.

3. Servicio de Cardiología y Unidad de Hemodinámica. Complejo Hospitalario Universitario de Santiago de Compostela (CHUS). SERGAS. Universidad de Santiago de Compostela, Santiago de Compostela, 15706 A Coruña, Spain.

4. División de Endocrinología, Departamento de Medicina, Complejo Hospitalario Universitario de Santiago (CHUS) and Universidad de Santiago de Compostela (USC), Santiago de Compostela, Spain.

5. CIBER Fisiopatologia de la Obesidad y Nutricion (CIBERobn), Madrid, Spain.

**Supplementary material**

**Figure S1.** Influence of previous heart failure (HF, **a** - **b**), previous acute myocardial infarct (AMI, **c** - **d**) or incidence of cardiovascular events (death or HF hospitalization, **e** - **f**) in the plasma levels of AGEs (**a**, **c** and **e**) and sRAGE (**b**, **d** and **f**) at time of discharge and after 1 or 6 months. Boxes represent interquartile ranges with median as horizontal line. Vertical bars demarcate the maximum to minimum range. * *p* < 0.05 vs. basal values, # < 0.05 vs. 1 month levels, by Wilcoxon test.


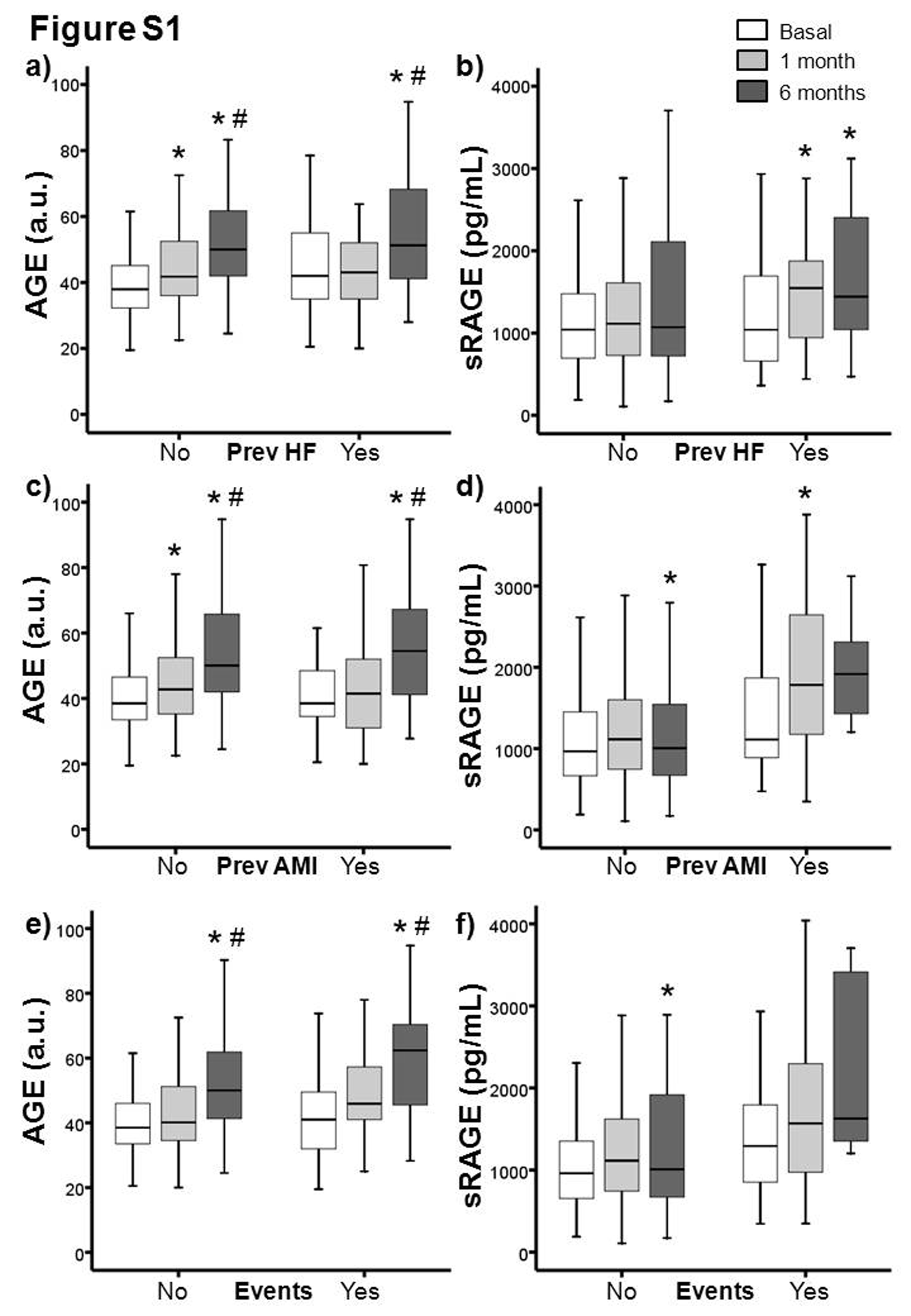


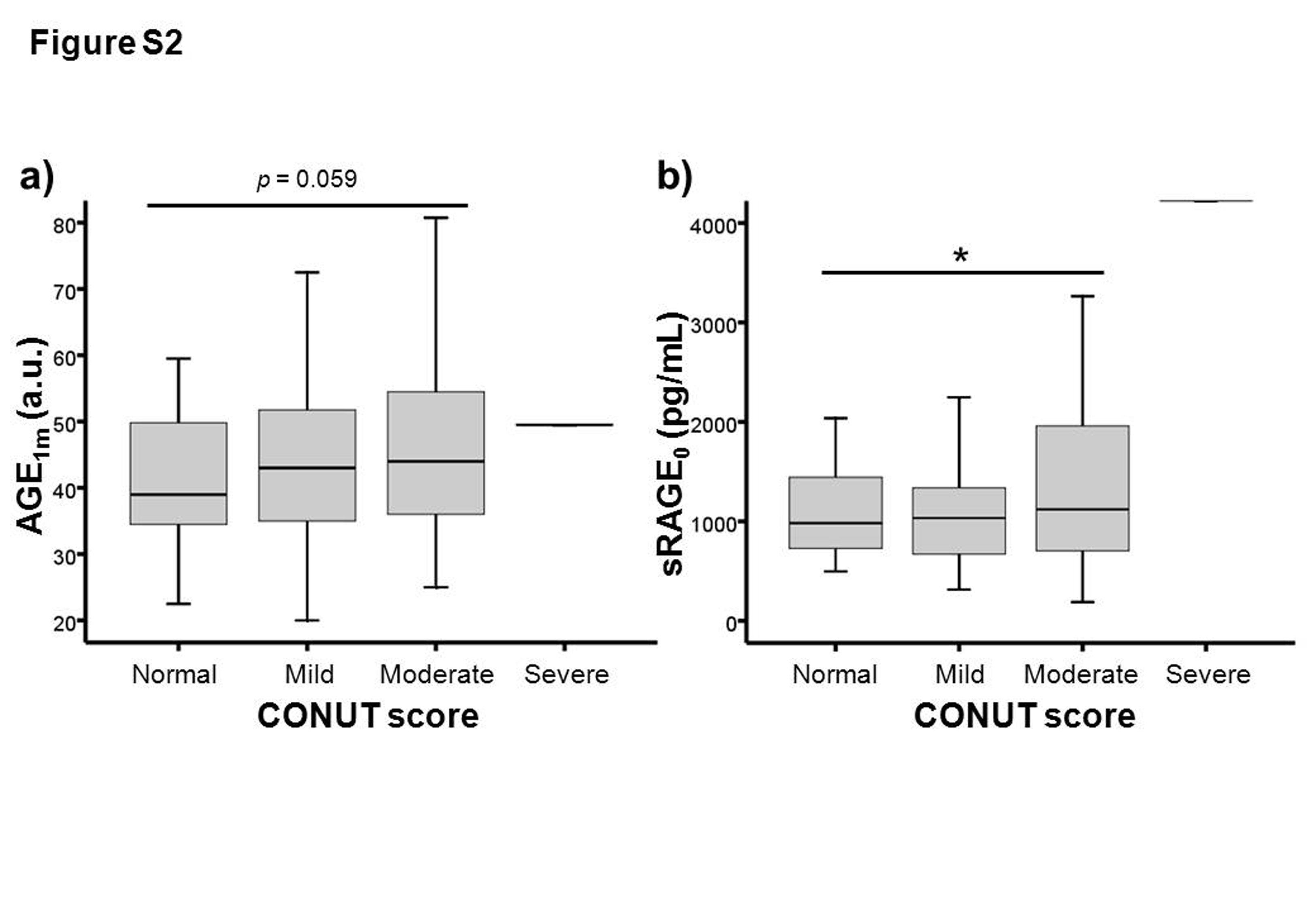


**Figure S2.** Levels of AGE at 1 month after discharge (**a**) and of sRAGE at discharge (**b**) in relation with the CONUT score at discharge. Boxes represent interquartile ranges with median as horizontal line. Vertical bars demarcate the maximum to minimum range. * *p* < 0.05 by Kruskal-Wallis test.

**Table S1**. Basal levels of biochemical parameters analysed. Data are presented as median [interquartile range] for the total population and grouped by the presence of events (death or HF hospitalization).

| **Biomarker (pg/mL)** | **Total population**  *n*=150 | **Events (-)**  *n*=111 | **Events (+)**  *n*=39 | ***p* value** |
| --- | --- | --- | --- | --- |
| C-peptide | 3262.1 [2174.7-4368.0] | 3354.3 [2370.4-4367.6] | 3181.3 [2044.0-4508.2] | 0.581 |
| Ghrelin | 1038.0 [678.1-1683.4] | 1047.6 [693.3-1683.4] | 877.5 [572.8-1676.1] | 0.215 |
| GIP | 638.3 [373.2-1039.7] | 633.3 [367.6-1015.4] | 649.2 [389.9-1186.4] | 0.711 |
| GLP-1 | 218.3 [185.4-411.9] | 212.5 [180.9-312.8] | 241.4 [204.6-491.8] | **0.022** |
| Glucagon | 231.6 [174.3-333.0] | 216.8 [166.0-308.4] | 284.9 [219.4-355.4] | **0.013** |
| Insulin | 618.2 [312.3-1074.2] | 605.7 [312.5-1108.0] | 648.9 [240.6-1036.8] | 0.570 |
| Leptin | 4454.9 [2348.6-8924.4] | 4255.2 [2229.0-9596.5] | 4632.8 [2668.0-7163.6] | 0.928 |
| PAI-1 | 13126.6 [9254.1-18881.0] | 13339.0 [9264.8-20190.1] | 12004.2 [9182.1-16087.4] | 0.356 |
| Resistin | 4632.3 [3465.5-7223.0] | 4536.0 [3265.0-6774.0] | 4968.1 [4224.4-8606.2] | 0.168 |
| Visfatin | 2928.5 [2329.9-3981.5] | 2919.1 [2255.3-3810.6] | 3040.2 [2378.2-4408.5] | 0.342 |
| **Abbreviations**: GIP: glucose-dependent insulinotropic polypeptide; GLP-1: glucagon-like peptide-1; PAI-1: plasminogen activator inhibitor-1. | | | | |

**Table S2**. Basal levels of biochemical parameters analysed. Data are presented as median [interquartile range] for the total population and grouped by the levels of AGE_1m_ and by sRAGE_0_.

| **Biomarker (pg/mL)** | **AGE_1m_ < 40 a.u.**  *n*=51 | **AGE_1m_ > 40 a.u**  *n*=73**.** | ***p* value** | **sRAGE_0_ < 1000 pg/mL**  *n*=73 | **sRAGE_0_ > 1000 pg/mL**  *n*=76 | ***p* value** |
| --- | --- | --- | --- | --- | --- | --- |
| C-peptide | 3011.9 [2164.1-3667.5] | 3767.0 [2362.5-4478.0] | **0.008** | 3519.6 [2468.3-4368.0] | 3181.3 [2057.6-4363.5] | 0.307 |
| Ghrelin | 923.8 [644.0-1535.1] | 1140.6 [758.1-1848.1] | 0.057 | 974.6 [646.9-1552.2] | 1107.2 [719.7-1821.3] | 0.205 |
| GIP | 627.3 [424.7-917.4] | 753.2 [431.0-1228.6] | 0.180 | 682.9 [377.4-1056.0] | 609.8 [360.9-1038.7] | 0.692 |
| GLP-1 | 253.6 [201.4-489.3] | 213.1 [180.8-436.1] | 0.083 | 246.7 [189.1-460.4] | 212.4 [182.9-358.3] | 0.197 |
| Glucagon | 287.5 [182.7-382.4] | 228.9 [174.5-315.2] | 0.059 | 242.6 [175.2-333.8] | 227.1 [170.0-330.8] | 0.509 |
| Insulin | 694.5 [358.1-1177.3] | 647.2 [258.7-1063.3] | 0.327 | 833.6 [364.9-1208.3] | 479.8 [294.8-917.0] | **0.019** |
| Leptin | 5060.1 [2735.4-11723.4] | 3830.8 [2119.0-8498.5] | 0.141 | 4606.9 [2648.4-10655.3] | 4367.1 [1983.4-7392.6] | 0.238 |
| PAI-1 | 15079 [11227-20322] | 12004 [8585-17381] | **0.046** | 13679.6 [8338.7-19324.6] | 12770.6 [9760.9-18653.4] | 0.997 |
| Resistin | 4224.4 [2911.9-5977.5] | 4938.1 [3835.2-7609.8] | **0.014** | 4444.8 [3263.2-6741.8] | 4822.1 [3729.2-7408.8] | 0.257 |
| Visfatin | 3080.1 [2367.5-4012.7] | 2834.5 [2276.6-3829.2] | 0.412 | 2883.9 [2171.0-3821.8] | 2947.0 [2364.1-4205.9] | 0.197 |
| Adiponectin (µg/mL) | 6.72 [4.85-12.44] | 7.57 [4.62-11.28] | 0.535 | 6.73 [4.64-10.15] | 7.14 [4.94-12.41] | 0.365 |
| **Abbreviations**: AGE_1m_: AGEs levels 1 month after discharge; GIP: glucose-dependent insulinotropic polypeptide; GLP-1: glucagon-like peptide-1; PAI-1: plasminogen activator inhibitor-1; sRAGE_0_: basal sRAGE levels. | | | | | | |
